# Rhizosecretion of stele-synthesized glucosinolates and their catabolites requires GTR-mediated import in Arabidopsis

**DOI:** 10.1093/jxb/erw355

**Published:** 2016-10-04

**Authors:** Deyang Xu, Franziska S Hanschen, Katja Witzel, Sebastian J Nintemann, Hussam Hassan Nour-Eldin, Monika Schreiner, Barbara Ann Halkier

**Affiliations:** 1 DynaMo Center, Department of Plant and Environmental Sciences, Faculty of Science, University of Copenhagen, Thorvaldsensvej, Frederiksberg C, Denmark; 2 Department of Plant Quality, Leibniz Institute of Vegetable and Ornamental Crops, Theodor-Echtermeyer-Weg, Grossbeeren, Germany; 3 Department of Plant Health, Leibniz Institute of Vegetable and Ornamental Crops, Theodor-Echtermeyer-Weg, Grossbeeren, Germany

**Keywords:** Arabidopsis, biosynthesis, exudate, glucosinolates (GLS), glucosinolates catabolites, GTR, import, rhizosphere, rhizosecretion, stele-synthesized phytochemicals, trans-cellular transport

## Abstract

Casparian strip-generated apoplastic barriers not only control the radial flow of both water and ions but may also constitute a hindrance for the rhizosecretion of stele-synthesized phytochemicals. Here, we establish root-synthesized glucosinolates (GLS) are in Arabidopsis as a model to study the transport routes of plant-derived metabolites from the site of synthesis to the rhizosphere. Analysing the expression of GLS synthetic genes in the root indicate that the stele is the major site for the synthesis of aliphatic GLS, whereas indole GLS can be synthesized in both the stele and the cortex. Sampling root exudates from the wild type and the double mutant of the GLS importers GTR1 and GTR2 show that GTR-mediated retention of stele-synthesized GLS is a prerequisite for the exudation of both intact GLS and their catabolites into the rhizosphere. The expression of the GTRs inside the stele, combined with the previous observation that GLS are exported from biosynthetic cells, suggest three possible routes of stele-synthesized aliphatic GLS after their synthesis: (i) GTR-dependent import to cells symplastically connected to the cortical cells and the rhizosphere; (ii) GTR-independent transport via the xylem to the shoot; and (iii) GTR-dependent import to GLS-degrading myrosin cells at the cortex. The study suggests a previously undiscovered role of the import process in the rhizosecretion of root-synthesized phytochemicals.

## Teaser

Implementing new methodology for sampling rhizosecreted glucosinolates from Arabidopsis, we discovered that import from apoplast is a prerequisite for the translocation of stele-synthesized phytochemicals across the endodermis barrier and into the rhizosphere.

## Introduction

The rhizosphere, the zone closely surrounding the roots where the exchange of micronutrients and organic matter occurs, is pivotal for both symbiotic and pathogenic interactions between the plant and soil organisms. There is an increased awareness of the importance of root exudation for the establishment of the microbial community in the rhizosphere (Bais *et al.*, 2006; Badri *et al.*, 2009*b*; Baetz and Martinoia, 2014; Carvalhais *et al.*, 2015) and in the response to environmental signals (Hoehenwarter *et al.*, 2016; Ziegler *et al.*, 2016). Depending on the species, the rhizosphere is apoplastically isolated from the rest of the root by the endodermis and/or exodermis with their Casparian strip-impregnated cells. Compounds synthesized outside or within the endodermis and/or exodermis can be released to the rhizosphere by passive diffusion or through specific exporters (Badri *et al.*, 2009*a*; Weston *et al.*, 2012). Compounds that are synthesized and released into the apoplast behind the apoplastic barriers (endodermis or exodermis) can follow one of two transport routes from the inside to the outside of the endodermal/exodermal cells. The first route is mediated by plasmodesmata (PD) connecting biosynthetic cells symplastically to the endodermis from which they are released by exporters to the rhizosphere. The second route is trans-cellular and involves the repeated efflux and influx through consecutively arranged and polarized carrier proteins (Andersen *et al.*, 2015). Even though several transporters have been identified for releasing compounds from root cells, these are all involved in the efflux of metabolites from cells outside the Casparian strip (Sugiyama *et al.*, 2007; Badri *et al.*, 2009*a*; Fourcroy *et al.*, 2014; Sasse *et al.*, 2015). By contrast, little is known about the transport route to the rhizosphere of specialized metabolites synthesized within the stele.

*Arabidopsis thaliana* (hereafter Arabidopsis) with glucosinolates (GLS) as major defence compounds has become an ideal model system to study the mechanism of transport of specialized metabolites because of its powerful genetic toolbox (Jørgensen *et al.*, 2015). Arabidopsis produces tryptophan-derived indole GLS and methionine-derived aliphatic GLS which are further divided into short-chained (with three to five methylene groups, C3–C5) and long-chained (with six to eight methylene groups, C6–C8) aliphatic GLS (Grubb and Abel, 2006), of which the latter are primarily synthesized and stored in the roots (Andersen *et al.*, 2013). Previous studies showed that root cells synthesizing long-chained aliphatic GLS by default export these GLS to the apoplast from where the two plasma-membrane localized glucosinolate importers, GTR1 and GTR2 (Nour-Eldin *et al.*, 2012), are suggested to import them into local storage cells (Andersen *et al.*, 2013). Indeed, *gtr1 gtr2* roots contain drastically reduced levels of long-chained, aliphatic GLS whereas rosettes over-accumulate them. This over-accumulation is caused by translocation of the exported GLS via the xylem (Andersen *et al.*, 2013; Madsen *et al.*, 2014). However, the total levels of GLS in *gtr1 gtr2* roots and rosettes are higher compared with wild-type levels. This shows that, in the absence of GTR1 and GTR2 which are both expressed in the root, higher amounts of GLS remain within the plant. It is conceivable that these higher amounts of GLS accumulating in the *gtr1 gtr2* mutant were destined for rhizosecretion. This hypothesis proposes that GTR-mediated GLS import plays an important role in the exudation pathway of stele-synthesized GLS. Noticeably, the tissue-specific localization of GLS biosynthesis has not previously been determined in Arabidopsis roots using non-invasive approaches. In addition, although exudation of the GSLs and/or their catabolites has been observed (Schreiner *et al.*, 2011) and suggested in *Brassica* crops (Choesin and Boerner, 1991; McCully *et al.*, 2008), intact GLS have not previously been identified in the root exudates of the model plant Arabidopsis, whereas biosynthetic intermediates and catabolites of GLS have been detected in different metabolomics studies (Badri *et al.*, 2013; Strehmel *et al.*, 2014, 2016; Monchgesang *et al.*, 2016; Ziegler *et al.*, 2016).

It is shown here that long-chained, aliphatic GLS are synthesized in the stele of Arabidopsis roots and exuded into the rhizosphere. A new sampling method has enabled the detection of both intact GLS and their catabolites in Arabidopsis root exudates. Therefore, we propose that elucidating the transport pathway of long-chained, aliphatic GLS provides a model system for understanding the root exudation of stele-synthesized compounds in general. As a first step towards investigating the transport route of GLS from the site of synthesis to the rhizosphere, we compared root exudates of *gtr1 gtr2* with the wild type and determined the localization of GTR at the cellular level. Our data indicate that GTR1 and GTR2 import into the symplasm is a prerequisite for the translocation of stele-synthesized GLS across the apoplastic barrier of the endodermis into the rhizosphere.

## Materials and methods

### Plant growth and sampling root exudates

*A. thaliana* Col-0 and mutants with a T-DNA insertion in both GTR1 (At3g47960) and GTR2 (At5g62680; *gtr1 gtr2* dKO) were grown on a non-sterile standardized plant growth substrate (Fruhstorfer Erde type P, Germany) with a pH value of 6.0 in a climate chamber under short-day conditions (8/16h light/ dark, 22 °C, 40–60% humidity). After 2 weeks, single plants were transferred to sand-filled pots and watered with nutrient solution as described by Gibeaut *et al.* (1997). After an additional 4 weeks, the sand was carefully removed from the roots and the plants were transferred to tubes filled with distilled water. After 1h, plants were transferred to fresh tubes (two plants per tube) filled with bi-distilled water and exudates were collected for 4h. In order to determine whether enzymatic hydrolysis of exuded GLS takes place, 100 µl of 0.1mM 2-propenyl GLS (Carl Roth GmbH, Germany) was added to each tube. During the whole procedure, plants were kept under the cultivation conditions. Exudates were filtered through a mixed cellulose ester membrane filter of pore size 0.22 µm (Carl Roth GmbH, Germany) to remove cellular debris and external microorganisms. Exudates from approximately 20 plants were pooled to give one sample and three samples were collected for each harvest. After collection of exudates, the roots were harvested and the fresh weight was determined to relate the amount of exudates to root biomass.

### Construct cloning

Derivatives of the USER™ compatible pCambia1300U and pCambia2300U plant expression vectors (Nour-Eldin *et al.*, 2006) were used in this work. The PCR product was then introduced into the PacI and Nt.BbvCI digested pCambia2300U vector by USER™ cloning. The mVenus variant of the yellow fluorescent protein was amplified with the same primer pair and introduced into the opened pCambia1300U vector.

The promoter sequences including upstream genomic sequences and the 5′ UTR of the *CYP83A1*, *CYP83B1* genes (1033bp and 1001bp, upstream of the start codon, respectively) were amplified from genomic DNA of *Arabidopsis thaliana* ecotype Columbia-0. The corresponding coding sequences were amplified from *Arabidopsis thaliana* ecotype Columbia-0 cDNA. The promoter and coding sequences were seamlessly fused by USER™ fusion (Geu-Flores *et al.*, 2007) and, at the same time, inserted into the USER™ cassette of the opened pCambia1300-mVenus (CYP83A1, CYP83B1) vectors. The proper fusion and insertion of sequences was confirmed by DNA sequencing.

### Cellular localization

Localization experiments were done on 12–14-d-old plants using a Leica SP5-X confocal microscope. Fluorophores were excited at the respective absorption maxima (514nm for YFP and 546nm for mOrange). Emission was collected at 525–540nm (YFP), and 560–580nm (mOrange).

### GUS assay and histology

Detection of GUS expression was performed with 5-bromo-4-chloro-3-indolyl-β-d-glucuronidase (X-Gluc). Tissues were vacuumed in 50mM sodium phosphate buffer, pH 7.0 (1% Triton X-100/1% DMSO/10mM EDTA/0.5mM potassium ferrocyanide/0.5mM potassium ferricyanide/1mM X-Gluc) and then incubated at 37 °C. The stained samples were washed twice with 70% ethanol, fixed in 4% formaldehyde, and cleared with 70% ethanol. Tissues were mounted in 50% glycerol, and microscopy was conducted. Histological analysis followed by sections was performed as described with some modifications. Briefly, tissues were gently fixed in 90% acetone, washed with water, incubated in 50mM sodium phosphate buffer, pH 7.0 (0.2% Triton X-100Z/10mM EDTA/10mM potassium ferrocyanide/10mM potassium ferricyanide/1mM X-Gluc), at 37 °C. The stained tissues were fixed in 4% paraformaldehyde, dehydrated with acetone then embedded in Spurr Resin. Resin sections (5 μm) were microtomed (Leica) and were observed after staining.

### GLS analysis

Desulfo-GLS profiles and concentrations of lyophilized plant tissue were determined as described before (by Witzel *et al.* (2015) with slight modifications: For the determination of GLS in exudates, the total exudate volume (500ml) was first subjected to catabolites analysis (see below). Then the exudate was spiked with 20 nmol of the internal standard (IST) 4-hydroxybenzyl GLS (Carl Roth GmbH, Karlsruhe, Germany) and loaded onto a 1ml DEAE-Sephadex A-25 ion-exchanger column (Sigma-Aldrich Chemie GmbH, Steinheim, Germany), further treated and analysed with the UHPLC-system as reported previously (Witzel *et al.*, 2015).

### Analysis of GLS breakdown products

For the determination of enzymatically formed breakdown products of the GLS in fresh plant tissues, benzonitrile (≥99.9%, Sigma-Aldrich Chemie GmbH) was added as an IST and catabolites were extracted with dichloromethane (Carl Roth GmbH; GC Ultra Grade) as described previously (Witzel *et al.*, 2015). For the analysis of glucosinolate breakdown products in exudates, the total volume of each exudate (500ml) was extracted with 50ml of dichloromethane in a separating funnel. The separated dichloromethane layer was dried using anhydrous NaSO_4_ (VWR International GmbH, Darmstadt, Germany; ≥99%) and the aqueous phase was re-extracted twice using 25ml of dichloromethane. The combined dichloromethane extract was dried under nitrogen to 300 µl and subjected to GC-MS analysis as described above.

### Statistical data analysis

To represent the changes in GLS levels, two-tailed Student’s *t* tests and one-way ANOVA followed by Tukey HSD Calculator multiple comparison *post-hoc* analysis (*P* <0.05) were performed using Origin Pro 2015.

### *In silico* analysis

Cell-type-specific expression was derived from microarray studies of RNA bound to ribosomes which were immunoprecipitated by use of epitope-tagged ribosomal protein from seedlings (Mustroph *et al.*, 2009; http://efp.ucr.edu/).

### Accession numbers

Sequence data from this article can be found in the Arabidopsis Genome Initiative or EMBL/GenBank data libraries under accession numbers: At3g47960 (GTR1); At5g62680 (GTR2); At AT4G13770 (CYP83A1); AT4G31500 (CYP83B1).

## Results

### Sampling of Arabidopsis root exudate

Towards our goal of understanding the root exudation process using Arabidopsi*s* and GLS as a model system, we developed a method to measure GLS and their catabolites in Arabidopsis root exudates under physiological conditions (see Supplementary Fig. S1A–C at *JXB* online). Based on previous reports, we optimized several steps in the procedure. First, we optimized root growth conditions by growing plants in sand instead of using a hydroponics system (Badri *et al.*, 2013; Strehmel *et al.*, 2014). Secondly, the exudation was carried out in de-ionized water instead of growth media. Thirdly, dichloromethane (CH_2_Cl_2_) was used to denature the myrosinase (Sah, 1999) while partitioning intact GLS to the aqueous phase and catabolites to the organic phase. Finally, we applied DEAE-Sephadex anion exchange chromatography to enrich GLS, followed by targeted HPLC and LC-MS detection methods to measure intact GLS (Brown *et al.*, 2003; Mikkelsen *et al.*, 2003).

We first tested the recovery efficiency of GLS in exudates by adding exogenous 2-propenyl GLS standard (a GLS not found in Arabidopsis Col-0) to the medium prior to sampling. We were able to recover 37±8% of the added 2-propenyl GLS while another 8±3% was recovered as the corresponding catabolite product allyl-ITC (AITC) (Supplementary Fig. S1D). As 2-propenyl GLS can be taken up and translocated from the root to the shoot (Francisco *et al.*, 2016), 2-propenyl GLS was analysed in the whole plant. No 2-propenyl GLS or AITC in either shoot or root tissue was detected (Supplementary Tables S1, S2). We concluded that, in the current sampling method, approximately 50% GLS was recovered from the exudate, of which approximately 75% GLS remained intact and approximately 25% was degraded during sample preparation.

### GLS and their catabolites in root exudates of the wild type

**Fig. 1.**
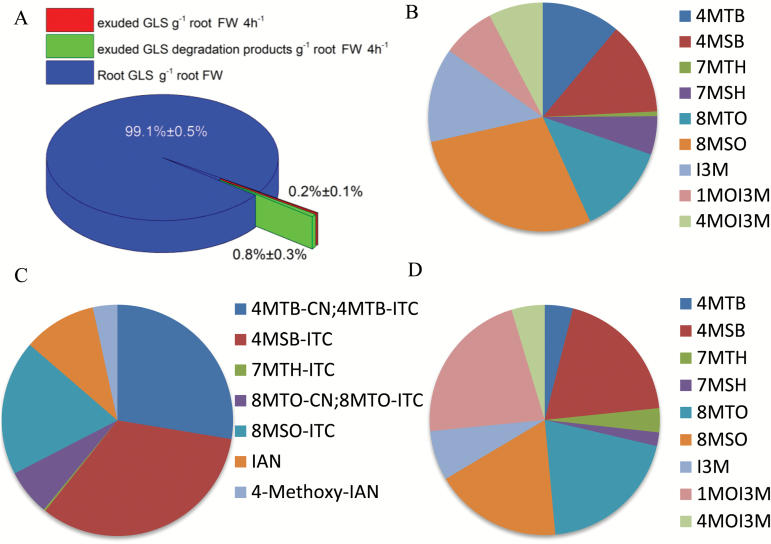
Profiling of GLS and their catabolites in Arabidopsis Col-0 root exudates. (A) Abundance of exuded GLS and catabolites per gram of root tissue collected in 4h. (B–D) Comparison of the profiles of GLS and their catabolites in different samples. The average percentage of each compound of at least two independent experiments are shown; for the details see Table 1. (B) GLS profile in the root exudate. (C) Profile of GLS catabolites in the root exudate. (D) GLS profile in the root.

Implementing our method, we set out to isolate both intact GLS and their catabolites in root exudates from Arabidopsis Col-0. After 4h, the total amount of collected GLS and their catabolites per gram of root tissue represented approximately 1% of GLS per gram of root tissue (Fig. 1A). The ratio of GLS to their catabolites was 1:4. In root exudates from the wild type, all three classes of GLS, i.e. short-chained and long-chained aliphatic GLS as well as indole GLS, were present (Fig. 1B; Table 1). The profile of aliphatic GLS in the root exudate was similar to the endogenous root profile, whereas the profile of indole GLS differed between the root and the exudate (Fig. 1B, D; Table 1). For several of the GLS catabolites, the profile in root exudates differed from the profile of the GLS in root exudates (Fig. 1B, C; Table 1) and from the catabolite profile of homogenized root tissue (Supplementary Table S2). The difference in profile of GLS and their catabolites between root and exudate (Fig. 1; Table 1) suggests that hydrolysis does not occur during sampling and extraction. Consistent with this observation, in the root exudate the ratio of catabolites to intact GLS is 4:1 while the ratio of catabolites to intact GLS for the exogenous 2-propenyl GLS is 1:4.

**Table 1.** Comparison of the profiles of GLS and their catabolites in Arabidopsis Col-0 roots and root exudates. Quantities are derived from the mean of at least two biological repeats each with three batches of plants (each *n*=20). GLS profiles of roots and root exudates, as well as the catabolite profile in root exudates were determined. To enable comparison between the amount of GLS and catabolites, the catabolites were converted into their corresponding GLS precursors. The values were determined by one-way ANOVA followed by Tukey HSD Calculator multiple comparison *post-hoc* analysis (*P* <0.05). The significance of differences between the profiles of roots and exudates in the respective comparisons are indicated by letters. Errors denote standard deviation. 4MTB, 4-(methylthio)butyl; 7MTH, 7-(methylthio)heptyl; 8MTO, 8-(methylthio)octyl; 3MSP, 3-(methylsulfinyl)propyl; 4MSB, 4-(methylsulfinyl)butyl; 7MSH, 7-(methylsulfinyl)heptyl; 8MSO, 8-(methylsulfinyl)octyl; I3M, 3-indolylmethyl; 1MOI3M, 1-methoxy-3-indolylmethyl; 4MOI3M, 4-methoxy-3-indolylmethyl glucosinolate. n.d. Not detected.

	Root	Exudate	Exudate
	GLS(%)^*a*^	GLS(%)^*a*^	GLS precursors of catabolites (%)^*b*^
Short-chained aliphatic GLS			
**4MTB**	3.9±1.5 a	11.1±8.8 a	27.6±9.2 b
**4MSB**	19.4±4.6 a	13.1±16.3 a	33.1±17.1 a
Long-chained aliphatic GLS			
**7MTH**	3.4±1.7 a	0.7±0.7 a	0.3±0.4 a
**7MSH**	1.9±1.6 a	5.4±2.9 b	n.d.
**8MTO**	19.9±8.9 a	12.9±11.5 a	6.4±8.4 b
**8MSO**	17.9±5.0 a	28.3±17.2 a	18.9±30.6 a
Indole GLS			
**I3M**	7.0±0.9 a	13.3±5.4 a	10.2±9.6 a
**1MOI3M**	21.9±7.7 a	7.5±6.3 b	n.d.
**4MOI3M**	4.7±2.2 a	7.7±5.9 a	3.4±5.9 a

^*a*^ Percentage of total GLS.

^*b*^ Percentage of total GLS catabolites.

### Cellular localization of the GLS synthesis enzymes CYP83A1 and CYP83B1 in the root

**Fig. 2.**
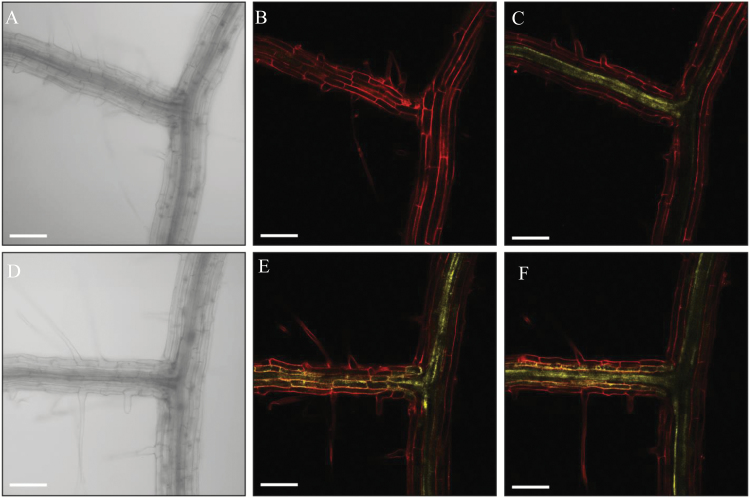
Propidium iodide-stained roots of 12-d-old Arabidopsis seedlings expressing *ProCYP83A1*:*CYP83A1-mVenus* (A–C) or *ProCYP83B1*:*CYP83B1-mVenus* (D–F). (A, D) Bright field channel. (B, E) Focused on the cortex layer of a lateral root branch point. (C, F) Focused on the stele tissue of a lateral root branch point. Scale bar: 100 μm.

In order to define the rhizosecretion route of GLS from the site of synthesis to the rhizosphere, we first analysed the root cell type-specific localization of the fluorophore-tagged biosynthetic enzymes CYP83A1 and CYP83B1 representative of aliphatic and indolic GLS biosynthesis, respectively (Naur *et al.*, 2003). We monitored fluorescence throughout a *z*-stack panel from the outer towards the inner layers of the root tissue. The CYP83A1-YFP signal was detected exclusively in the stele along the main root with relatively higher expression in the vasculature at the lateral root branch site (Fig. 2A–C). By contrast, the CYP83B1 signal was detected in both the outer and inner layers of root cells at both the main root and the lateral root branch sites (Fig. 2D–F). The stele-specific localization of aliphatic biosynthetic enzymes, combined with the presence of GLS in the rhizosphere, indicate that the transport route of aliphatic GLS from the site-of-synthesis tothe rhizosphere requires a coupled trans-cellular transport pathway and/or symplastic pathway to pass the Casparian strip. By contrast, a trans-cellular independent, but symplastic transport-dependent pathway may exist for the rhizosecretion of indole GLS.

### Role of GTR importers in the rhizosecretion of GLS

To explore the root exudation process of GLS, we investigated whether GTR1 and GTR2 play a role in this process. Previously, GTR1 and GTR2 have been shown to play a key role in the loading of GLS into seeds. In that study, there was no significant effect on GLS in seeds of the *gtr1* single mutants and only about a 50% reduction in the *gtr2* single mutants, whereas the *gtr1 gtr2* double mutants had zero GLS left in seeds (Nour-Eldin *et al.*, 2012). Due to redundancy in GTR1 and GTR2 function we have, in the present study, chosen to investigate *gtr1 gtr2* double mutants to maximize any observed effect on root exudation. We first analysed the GLS distribution pattern in the rosette and roots of 6-week-old wild type and *gtr1 gtr2* mutants. In *gtr1 gtr2,* we observed a dramatic reduction of aliphatic GLS in roots and an over-accumulation in the rosette (Supplementary Fig. S2; Table S1), in agreement with previous observations for 3-week-old hydroponically grown plants (Andersen *et al.*, 2013). This over-accumulation of root-synthesized GLS in the rosette suggested that, in the wild type, root storage cells and/or the rhizosphere (and not the rosette) were the major sinks for those GLS.

**Fig. 3.**
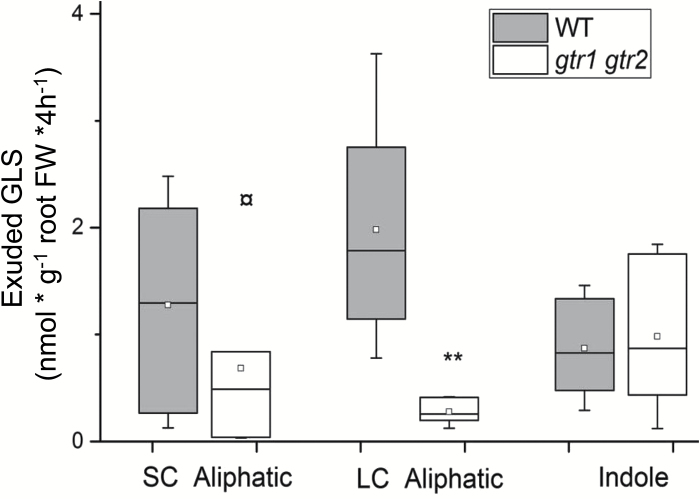
Exudation of GLS in the wild type (Col-0) and *gtr1 gtr2*. GLS exudates were collected after 4h in water with 6-week-old, sand-grown Arabidopsis wild-type (Col-0) and *gtr1gtr2* mutant plants. GLS are grouped into short-chained (SC) (C3–C5) and long-chained (LC) (C6–C8) aliphatic and indole GLS (Indole). For individual GLS data see Supplementary Table S1 and Table 1. The box is determined by the 25th and 75th percentiles. The whiskers are determined by the 5th and 95th percentiles. Median and mean are shown as a line and a square. ** Statistically significant differences of *gtr1 gtr2* compared with equivalent Col-0 parts (two-tailed Students *t* test, *P* <0.05).

When we measured the exudates from 6-week-old wild type and *gtr1 gtr2,* we found that exudates of *gtr1gtr2* root contained strongly reduced levels of particularly long-chained aliphatic GLS (Fig. 3; Table 2). The levels of 4MSB—the major short-chained aliphatic GLS—varied substantially between the different biological repeats, but was not significantly changed in *gtr1 gtr2* compared with the wild type, as was also observed for indole GLS (Table 2). This showed that exudation of stele-synthesized, long-chained aliphatic GLS requires a GTR-mediated import process of GLS from the sites of synthesis to a symplastic domain for exudation.

**Table 2.** GLS present in the exudates of Arabidopsis Col-0 and the *gtr1 gtr2* mutantThe quantities shown in nmol g^-1^ root fresh weight are derived from the mean of three biological experiments with three batches of plants (each *n*=20). Errors denote standard deviation. 4MTB, 4-(methylthio)butyl; 7MTH, 7-(methylthio)heptyl; 8MTO, 8-(methylthio)octyl; 3MSP, 3-(methylsulfinyl)propyl; 4MSB, 4-(methylsulfinyl)butyl; 7MSH, 7-(methylsulfinyl)heptyl; 8MSO, 8-(methylsulfinyl)octyl; I3M, 3-indolylmethyl; 1MOI3M, 1-methoxy-3-indolylmethyl; 4MOI3M, 4-methoxy-3-indolylmethyl glucosinolate. n.d. Not detected. Significant differences between Col-0 and *gtr1 gtr2* in the respective tissue comparisons are indicated by asterisks (* *P* <0.05).

	Col-0	*gtr1 gtr2*
Methylthioalkyl GLS		
4MTB	0.49±0.39	0.02±0.02*
7MTH	0.03±0.03	0.01±0.01
8MTO	0.57±0.51	0.07±0.10*
Methylsulfinylalkyl GLS		
4MSB	0.58±0.72	0.69±1.05
7MSH	0.24±0.13	0.01±0.02*
8MSO	1.25±0.76	0.23±0.15*
Indole GLS		
I3M	0.59±0.24	1.39±1.21
1MOI3M	0.33±0.28	0.66±0.55
4MOI3M	0.34±0.26	0.56±0.44

### Role of GTR importers in rhizosecretion of GLS catabolites

**Fig. 4.**
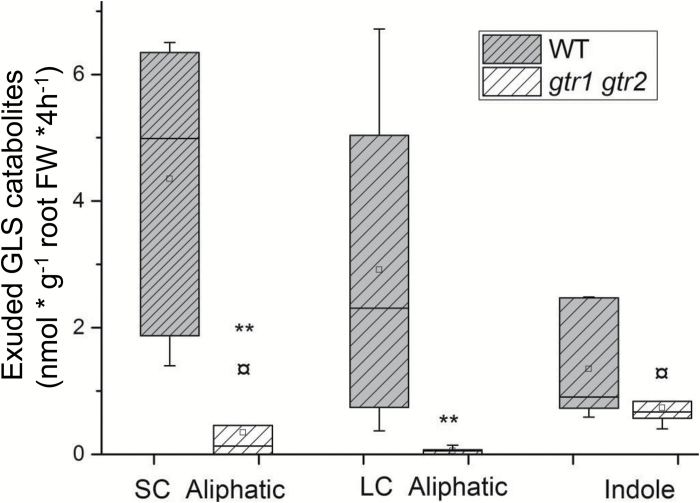
Exudation of GLS catabolites in wild-type (Col-0) and *gtr1 gtr2* plants. Exudate of GLS catabolites was collected after 4h in water with 6-week-old, sand-grown Arabidopsis wild-type (Col-0) and *gtr1 gtr2* plants. Catabolites of GLS are grouped according to their precursors of short-chained (SC) (C3–C5) and long-chained (LC) aliphatic (C6–C8) and indole (Indole) GLS. For individual GLS catabolite data see Table 2. The whiskers are determined by the 5th and 95th percentiles. Median and mean are shown as a line and a square. ** Statistically significant differences of *gtr1 gtr2* compared with equivalent Col-0 parts (two-tailed Students *t* test, *P* <0.05).

Catabolites of both short-chained and long-chained aliphatic GLS in root exudates in the *gtr1 gtr2* mutants constituted 10% of the concentration in wild-type exudates (Fig. 4; Table 3). Although GLS levels in the roots of *gtr1 gtr2* themselves were also decreased, the decrease was more pronounced for the catabolites than for intact GLS which suggests that GTR-mediated import into, for example, myrosin cells is a critical step prior to hydrolysis and exudation. Puzzlingly, a major part of total aliphatic GLS catabolites in root exudates of *gtr1 gtr2* mutants was from short-chained aliphatic GLS (Table 3) that was not detectable in the roots of *gtr1 gtr2* (Supplementary Table S1) proposing a complete hydrolysis of short-chained aliphatic GLS in the mutant. Catabolites of indole GLS were not significantly reduced in root exudates of *gtr1 gtr2* mutants compared with the wild type (Fig. 4; Table 2).

**Table 3.** GLS catabolites present in the exudates of Arabidopsis Col-0 and the *gtr1 gtr2* mutant Quantities shown in nmol g^-1^ root fresh weight are derived from the mean of three biological experiments with three batches of plants (each *n*=20). Errors denote standard deviation. 4MTB-CN, 5-(methylthio)pentylnitrile; 4MTB-ITC, 4-(methylthio)butyl ITC; 7MTH-ITC, 7-(methylthio)heptyl ITC; 8MTO-CN, 9-(methylthio)nonylnitrile; 8MTO-ITC, 8-(methylthio)octyl ITC; 4MSB-ITC, 4-(methylsulfinyl)butyl ITC; 8MSO-ITC, 8-(methylsulfinyl)octyl ITC; IAN, indole-3-acetonitrile; 4-Methoxy-IAN, 4-methoxyindole-3-acetonitrile; ITC, isothiocyanate. n.d. Not detected. Significant differences between Col-0 and the *gtr1 gtr2* mutant in the respective tissue comparisons are indicated by asterisks (* *P* <0.05).

	Col-0	*gtr1 gtr2*
Methylthioalkyl catabolites		
**4MTB-CN**	0.91±0.22	0.003±0.005*
**4MTB-ITC**	1.19±0.48	n.d.
**7MTH-ITC**	0.02±0.03	n.d.
**8MTO-CN**	0.22±0.38	n.d.
**8MTO-ITC**	0.27±0.26	0.02±0.04
Methylsulfinylalkyl catabolites		
**4MSB-ITC**	2.52±1.30	1.53±2.54
**8MSO-ITC**	1.44±2.33	n.d.
Indole catabolites		
**IAN**	0.78±0.73	0.31±0.27
**4-Methoxy-IAN**	0.26±0.45	n.d.

### Cellular expression of GTR2 in the root

**Fig. 5.**
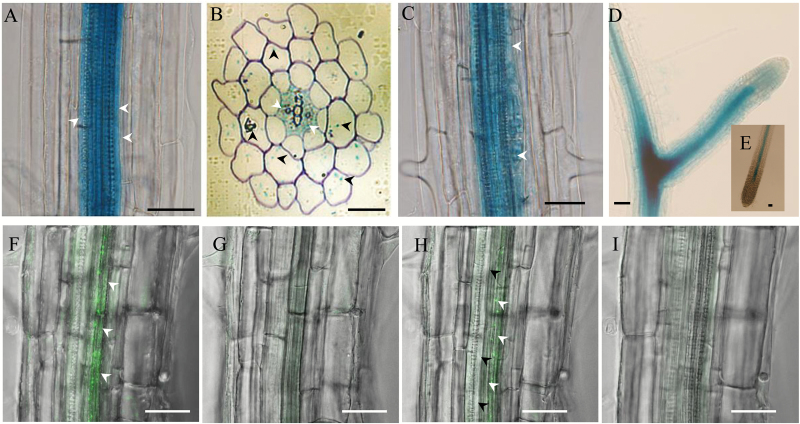
Cellular localization of GTR2 in the root. Transgenic Arabidopsis plants expressing a GTR2-mOrange fusion and GFP-GUS fusion under the control of a 2-kb promoter of GTR2 were analysed by confocal microscopy or GUS staining. (A) GUS signal shown in pericycle cells (white arrowheads) and the inner cell layers. (B) Cross-section of a root region similar to the one shown in (A). GUS signals in the stele (white arrowheads) and sporadically distributed in the cortex and epidermal cells (black arrowheads). (C) GUS signals in a lateral root initiation site (arrowheads showed lateral root founder cells). (D) GUS signals in a lateral root mature stage. (E) GUS signal in the main root tip. (f–I) mOrange signal from longitudinal optical sections along the *x-y* axis of intact roots of GTR2-mOrange. The mOrange signals are in green. (F) *Z*-stack of optical sections of intact roots of GTR2-mOrange. (G–I) Serial optical sections along the *z*-axis from the top to the bottom of the same region of root as in (F). The GTR2-mOrange was predominantly expressed in cells next to proxylem cells (F, H, white arrowheads) but not in proxylem cells (I, black arrowheads). Bars in (A, C-E and F–I)=25 μm. Bars in (B)=100 μm.

In order to investigate the localization of GTR-mediated import for stele-synthesized aliphatic GLS, we analysed the cell type-specific expression of GTR2. In agreement with microarray and translatome databases (Mustroph *et al.*, 2009), a previous study indicated that GTR1 and GTR2 have similar tissue expression patterns in the root (Andersen *et al.*, 2013). Thus, we used GTR2 reporter lines for further analysis. We first analysed the cellular expression patterns by promoter GUS fusion in Arabidopsis root (Nour-Eldin *et al.*, 2012). Promoter activity was detected at the pericycle and inner cell layers in the stele of the elongation and maturation regions (Fig. 5A, B, arrows). Cross-sections of the root within the same region confirmed the stele accumulation and also indicated weak *GTR2* expression in the cortical and epidermal cells (Fig. 5B, arrow heads). In the lateral root initiation region, *GTR2* was expressed in lateral root founder cells (Fig. 5C, arrow heads), which are differentiated from specific pericycle cells from the xylem pole. At a later developmental stage of the lateral roots, the signal spread to the whole root primordia (stage III–V) and accumulated at later root branch sites (Fig. 5D). In the mature lateral root it was restricted to the vasculature and no signal was detected in the endodermal cells and root tip of the mature roots (Fig. 5A–E). Next, we analysed the cellular distribution of GTR2 protein using fluorescent protein fusion (Andersen *et al.*, 2013). In agreement with the GUS signal, in the main root of Arabidopsis seedlings GTR2-mOrange signals were detected in the stele (Fig. 5F, G–I), of which cells next to pro-xylem cells (Fig. 5F, H) showed the strongest expression. The expression of the GTRs inside the stele (including the pericycle cells), and sporadically in the cortex, shows where the import of GLS occurs prior to exudation.

## Discussion

### Rhizosecretion of GLS and their catabolites in Arabidopsis

One prerequisite for using GLS in Arabidopsi*s* as a model system for studying root exudation is that GLS are exuded and detectable. Accordingly, we developed a method that enables the detection of intact GLS and their catabolites simultaneously in root exudates from Arabidopsis. Previous studies indicated that other plants within the Brassicaceae family can exude both intact GLS and their catabolites (Schreiner *et al.*, 2011; Ngala *et al.*, 2015). Hitherto, in Arabidopsis, however, only GLS catabolites and GLS biosynthetic intermediates were identified in root exudates (Badri *et al.*, 2013; Strehmel *et al.*, 2014, 2016; Monchgesang *et al.*, 2016). In our method, we collected root exudates from sand-grown plants in de-ionized water instead of nutrient medium which was previously used for hydroponically cultured plants (Badri *et al.*, 2013; Strehmel *et al.*, 2014). By replacing the artificial hydroponic growth conditions with sand, we reduced the hypoxia stress often seen in insufficiently aerated hydroponics. Furthermore, we collected the exudate from plants in de-ionized water instead of nutrient medium, thereby reducing the amount of salt in the samples. The previously-reported desalination step may have caused the loss of GLS and be the reason why intact GLS are rarely (if at all) detected in root exudate analyses (Strehmel *et al.*, 2014). In addition, the previous inability to detect intact GLS in exudates may be caused by myrosinase-mediated hydrolysis during the isolation process. The use of dichloromethane (CH_2_Cl_2_) instead of AgNO_3_ (Schreiner *et al.*, 2011)—to inactivate myrosinase and simultaneously partition intact GLS to the aqueous phase and catabolites to the organic phase—appears to represent an improvement. Based on the proportion of recovered intact exogenous 2-propenyl GLS, we estimate that approximately 20% of the total exuded GLS is degraded during the 4h of sampling.

It is noticeable that the profiles of GLS and GLS catabolites in root exudates differed significantly (Fig.1; Table 1), in spite of the large variance of individual GLS between biological repeats (Table 1). Assuming that GLS are hydrolysed at the same rate, our data suggest that hydrolysis does not occur during sampling and extraction, as this would have given the same profile for intact and hydrolysed GLS in the root exudate. All three classes of GLS and their catabolites, i.e. short-chained and long-chained aliphatic GLS as well as indole GLS, were present (Table 1) which, to the best of our knowledge, is the first time this has been reported.

The total amount of GLS and catabolites collected in wild-type root exudate after 4h represented approximately 1% of GLS per gram of root tissue (Fig. 1A). Hence, over a longer time, Arabidopsis roots may exudate a considerable amount of GLS and/or their catabolites into the rhizosphere where a vast diversity of plant-associated microbiota live. PEN2-dependent indole glucosinolate metabolism is required for the plant growth promotion effect by the root endophyte *Colletotrichum tofieldiae* in phosphate-limited conditions (Hiruma *et al.*, 2016). Despite substantial knowledge about the defensive roles of GLS, the physiological significance of their role in the rhizosphere is largely unknown. The measurement of GLS in root exudates of the model plant Arabidopsis is a tool for dissecting the role of GLS in plant–rhizosphere interactions.

### Import-dependent rhizosecretion of stele-synthesized metabolites

A key finding in this study is that GTR1- and GTR2-mediated import is critical for the rhizosecretion of stele-synthesized GLS. Intuitively, one would have anticipated that the rhizosecreted phytochemicals were produced in the outer cell layers of the root rather than in the central stele. The formation of GLS in the stele might benefit from the carbon supply from leaf-active primary metabolism via the phloem. Concurrently, the vascular-associated synthesis followed by export to the apoplast enables the efficient mobilization of defence compounds via the ascending xylem, in addition to loading on to the endodermis symplastically for exudation. This suggests that the GLS importers GTR1 and GTR2 are essential in orchestrating the flow of stele-synthesized GLS to balance above- and below-ground defence in response to environmental challenge. Many other defence compounds like nicotine (Morita *et al.*, 2009), tropane alkaloids (Ziegler and Facchini, 2008), and pyrrolizidine alkaloids (Hartmann and Ober, 2000) are synthesized in roots and stored in leaves. Many of these compounds are also involved in rhizosphere communication (Zhuang *et al.*, 2013; Tsednee *et al.*, 2014). This suggests that modulating specific transport activities (in our case, plasma-membrane importers) are essential for shaping the distribution pattern of the defence compounds at the organismal level, for example, between shoot, root, and rhizosphere.

In contrast to the stele-specific synthesis of long-chained aliphatic GLS, indole GLS are synthesized in both the stele and cortex and their rhizosecretion occurs in a GTR1- and GTR2-independent manner (Figs 2, 3). As GTR1 and GTR2 show broad substrate specificity towards GLS (Nour-Eldin *et al.*, 2012), the presence of indole GLS at similar levels in root exudates of *gtr1 gtr2* and the wild type suggests that an additional and indole-specific GLS importer(s) exist which functions redundantly with GTR1 and GTR2.

The similar level of exuded 4MSB from *gtr1 gtr2* mutant and wild-type roots (Fig. 4; Table 2) suggests that exudation of this glucosinolate is not dependent on GTR transport activity. Interestingly, 4MSB was detected inside cortical cells in a cell type-specific metabolite profiling of root cells (Moussaieff *et al.*, 2013) and expression of 4MSB-specific biosynthesis genes, for example, *CYP79F2* and *FMOGS-OX1*, includes cortical cells (Supplementary Fig. S3B) (Chen *et al.*, 2003; Hruz *et al.*, 2008; Li *et al.*, 2011). Therefore, we suggest that root-synthesized 4MSB is continuously exported to the rhizosphere from cells outside the Casparian strip which agreed with the GLS distribution in canola roots (*Brassica napus*) by cell-specific sulphur analyses (McCully *et al.*, 2008).

Exudation of catabolites of aliphatic GLS relies on GTR1 and GTR2 activity. A possible scenario is that the GTRs import aliphatic GLS to myrosin cells for hydrolysis prior to exudation. The expression pattern of the major root myrosinases TGG4 and TGG5 (Supplementary Fig. S3C, D) overlaps with that of GTR1 and GTR2 in the cortex (data not shown and Fig. 5B). This supports the loading of GLS into myrosin cells by GTRs.

**Fig. 6.**
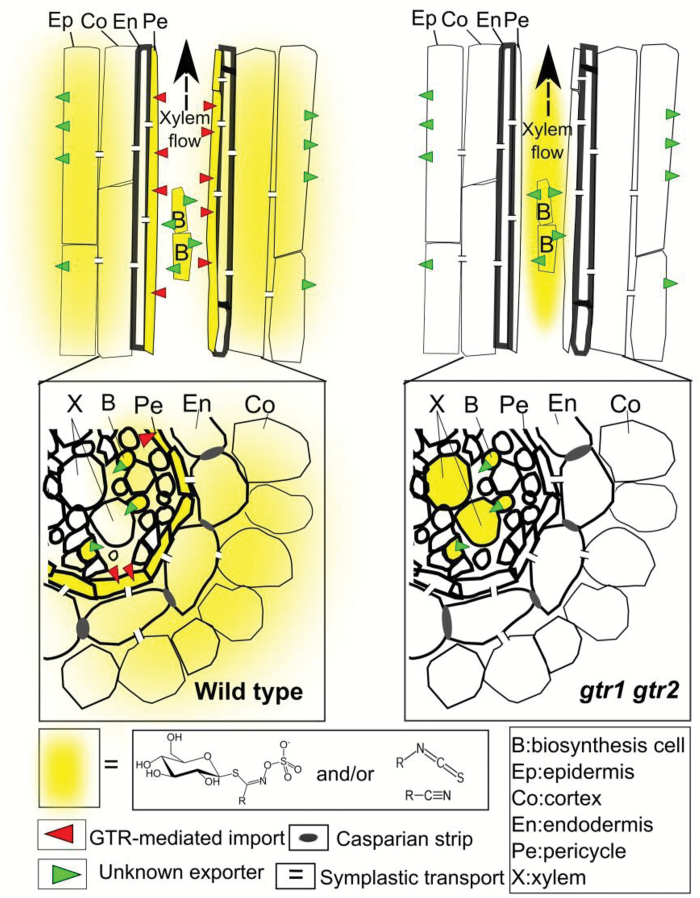
Model for the transport route of stele-synthesized GLS and their catabolites to the rhizosphere in Arabidopsis. Upon synthesis in the cells adjacent to the root vasculature, aliphatic GLS are exported out of the biosynthetic cells by unknown exporter(s). A GTR1- and GTR2-dependent uptake of aliphatic GLS from the apoplast is likely to occur in cells adjacent to the xylem, For example, in the pericycle cells which are symplastically connected with the endodermal cells for exudation, as well as into phloem companion cells for long-distance transport. From endodermal cells, GLS are either exported by an unknown transporter to the cortex and rhizosphere or enter a symplastic route toward the rhizosphere via plasmodesmata. GTRs are essential for GLS entering myrosin cells which produce GLS catabolites for secretion by unknown mechanism. The GLS that are not taken up by GTRs are translocated to the shoot through the xylem. In *gtr1 gtr2* mutants, the reduced levels of GLS in root exudates are due to a defect in the loading of GLS into the symplastic domain for exudation in the mutant. A consequence of the lack of this import is the translocation of the apoplastic localized, stele-synthesized GLS into the shoot through ascending xylem sap and thus a reduction of root GLS and an over accumulation of shoot GLS in the mutant.

In summary, our data indicate that two import processes are critical for rhizosecretion: (i) the import of stele-synthesized long-chained aliphatic GLS into an endodermis-connected symplastic domain, and (ii) the import of short-chained aliphatic GLS from the apoplast in the cortex into myrosin cells (Fig. 6). This is an important first step in understanding the root exudation process. Future studies will reveal how intact GLS and their catabolites are exported from root cells.

## Supplementary data

Supplementary data can be found at *JXB* online.

Figure S1. Sampling and profiling of GLS and their catabolites in Arabidopsis root exudates.

Figure S2. GLS concentration of rosettes and roots of 6-week-old sand-grown Arabidopsis wild type (Col-0) and *gtr1gtr2*.

Figure S3. Cell type-specific expression of *MAM3, FMOGS-OX1, TGG4*, and *TGG5* in Arabidopsis roots.

Table S1. GLS in the leaf and root of Arabidopsis Col-0 and the *gtr1 gtr2* mutant.

Table S2. GLS catabolites in the leaf and root of Arabidopsis.

## Supplementary Material

supplementary_figures_S1_S3_tables_S1_S2

## References

[CIT0001] Andersen TG Barberon M Geldner N 2015 Suberization–the second life of an endodermal cell. Current Opinion in Plant Biology 28, 9–15. 26343015 10.1016/j.pbi.2015.08.004

[CIT0002] Andersen TG Nour-Eldin HH Fuller VL Olsen CE Burow M Halkier BA 2013 Integration of biosynthesis and long-distance transport establish organ-specific glucosinolate profiles in vegetative Arabidopsis. The Plant Cell 25, 3133–3145. 23995084 10.1105/tpc.113.110890 PMC3784604

[CIT0003] Badri DV Chaparro JM Zhang R Shen Q Vivanco JM 2013 Application of natural blends of phytochemicals derived from the root exudates of Arabidopsis to the soil reveal that phenolic-related compounds predominantly modulate the soil microbiome. The Journal of Biological Chemistry 288, 4502–4512. 23293028 10.1074/jbc.M112.433300 PMC3576057

[CIT0004] Badri DV Quintana N El Kassis EG Kim HK Choi YH Sugiyama A Verpoorte R Martinoia E Manter DK Vivanco JM 2009 *a* An ABC transporter mutation alters root exudation of phytochemicals that provoke an overhaul of natural soil microbiota. Plant Physiology 151, 2006–2017. 19854857 10.1104/pp.109.147462 PMC2785968

[CIT0005] Badri DV Weir TL van der Lelie D Vivanco JM 2009 *b* Rhizosphere chemical dialogues: plant-microbe interactions. Current Opinion in Biotechnology 20, 642–650. 19875278 10.1016/j.copbio.2009.09.014

[CIT0006] Baetz U Martinoia E 2014 Root exudates: the hidden part of plant defense. Trends in Plant Science 19, 90–98. 24332225 10.1016/j.tplants.2013.11.006

[CIT0007] Bais HP Weir TL Perry LG Gilroy S Vivanco JM 2006 The role of root exudates in rhizosphere interations with plants and other organisms. Annual Review of Plant Biology 57, 233–266. 10.1146/annurev.arplant.57.032905.105159 16669762

[CIT0008] Brown PD Tokuhisa JG Reichelt M Gershenzon J 2003 Variation of glucosinolate accumulation among different organs and developmental stages of *Arabidopsis thaliana*. Phytochemistry 62, 471–481. 12620360 10.1016/s0031-9422(02)00549-6

[CIT0009] Carvalhais LC Dennis PG Badri DV Kidd BN Vivanco JM Schenk PM 2015 Linking jasmonic acid signaling, root exudates, and rhizosphere microbiomes. Molecular Plant–Microbe Interactions 28, 1049–1058. 26035128 10.1094/MPMI-01-15-0016-R

[CIT0010] Chen SX Glawischnig E Jorgensen K Naur P Jorgensen B Olsen CE Hansen CH Rasmussen H Pickett JA Halkier BA 2003 CYP79F1 and CYP79F2 have distinct functions in the biosynthesis of aliphatic glucosinolates in Arabidopsis. The Plant Journal 33, 923–937. 12609033 10.1046/j.1365-313x.2003.01679.x

[CIT0011] Choesin DN Boerner REJ 1991 Allyl isothiocyanate release and the allelopathic potential of *Brassica napus* (Brassicaceae). American Journal of Botany 78, 1083–1090.

[CIT0012] Fourcroy P Siso-Terraza P Sudre D Saviron M Reyt G Gaymard F Abadia A Abadia J Alvarez-Fernandez A Briat JF 2014 Involvement of the ABCG37 transporter in secretion of scopoletin and derivatives by *Arabidopsis* roots in response to iron deficiency. New Phytologist 201, 155–167. 24015802 10.1111/nph.12471

[CIT0013] Francisco M Joseph B Caligagan H Li B Corwin JA Lin C Kerwin R Burow M Kliebenstein DJ 2016 The defense metabolite, allyl glucosinolate, modulates *Arabidopsis thaliana* biomass dependent upon the endogenous glucosinolate pathway. Frontiers in Plant Science doi: 10.3389/fpls.2016.00774. 10.3389/fpls.2016.00774 PMC4887508 27313596

[CIT0014] Geu-Flores F Nour-Eldin HH Nielsen MT Halkier BA 2007 USER fusion: a rapid and efficient method for simultaneous fusion and cloning of multiple PCR products. Nucleic Acids Research doi: 10.1093/nar/gkm106 10.1093/nar/gkm106 PMC1874642 17389646

[CIT0015] Gibeaut DM Hulett J Cramer GR Seemann JR 1997 Maximal biomass of *Arabidopsis thaliana* using a simple, low-maintenance hydroponic method and favorable environmental conditions. Plant Physiology 115, 317–319. 9342857 10.1104/pp.115.2.317 PMC158488

[CIT0016] Grubb CD Abel S 2006 Glucosinolate metabolism and its control. Trends in Plant Science 11, 89–100. 16406306 10.1016/j.tplants.2005.12.006

[CIT0017] Hartmann T Ober D 2000 Biosynthesis and metabolism of pyrrolizidine alkaloids in plants and specialized insect herbivores. Biosynthesis: Aromatic Polyketides, Isoprenoids, Alkaloids 209, 207–243.

[CIT0018] Hiruma K Gerlach N Sacristan S 2016 Root endophyte *Colletotrichum tofieldiae* confers plant fitness benefits that are phosphate status dependent. Cell 165, 464–474. 26997485 10.1016/j.cell.2016.02.028 PMC4826447

[CIT0019] Hoehenwarter W Monchgesang S Neumann S Majovsky P Abel S Muller J 2016 Comparative expression profiling reveals a role of the root apoplast in local phosphate response. BMC Plant Biology doi: 10.1186/s12870-016-0790-8. 10.1186/s12870-016-0790-8 PMC4849097 27121119

[CIT0020] Hruz T Laule O Szabo G Wessendorp F Bleuler S Oertle L Widmayer P Gruissem W Zimmermann P 2008 Genevestigator v3: a reference expression database for the meta-analysis of transcriptomes. Advances in Bioinformatics doi: 10.1155/2008/420747. 10.1155/2008/420747 PMC2777001 19956698

[CIT0021] Jørgensen ME Nour-Eldin HH Halkier BA 2015 Transport of defense compounds from source to sink: lessons learned from glucosinolates. Trends in Plant Science 20, 508–514. 25979806 10.1016/j.tplants.2015.04.006

[CIT0022] Li J Kristiansen KA Hansen BG Halkier BA 2011 Cellular and subcellular localization of flavin-monooxygenases involved in glucosinolate biosynthesis. Journal of Experimental Botany 62, 1337–1346. 21078824 10.1093/jxb/erq369

[CIT0023] Madsen SR Olsen CE Nour-Eldin HH Halkier BA 2014 Elucidating the role of transport processes in leaf glucosinolate distribution. Plant Physiology 166, 1450–1462. 25209984 10.1104/pp.114.246249 PMC4226354

[CIT0024] McCully ME Miller C Sprague SJ Huang CX Kirkegaard JA 2008 Distribution of glucosinolates and sulphur-rich cells in roots of field-grown canola (*Brassica napus*). New Phytologist 180, 193–205. 18565145 10.1111/j.1469-8137.2008.02520.x

[CIT0025] Mikkelsen MD Petersen BL Glawischnig E Jensen AB Andreasson E Halkier BA 2003 Modulation of CYP79 genes and glucosinolate profiles in Arabidopsis by defense signaling pathways. Plant Physiology 131, 298–308. 12529537 10.1104/pp.011015 PMC166809

[CIT0026] Monchgesang S Strehmel N Schmidt S Westphal L Taruttis F Muller E Herklotz S Neumann S Scheel D 2016 Natural variation of root exudates in *Arabidopsis thaliana–*linking metabolomic and genomic data. Scientific Reports doi:10.1038/srep29033. 10.1038/srep29033 PMC4929559 27363486

[CIT0027] Morita M Shitan N Sawada K Van Montagu MCE Inzé D Rischer H Goossens A Oksman-Caldentey KM Moriyama Y Yazaki K 2009 Vacuolar transport of nicotine is mediated by a multidrug and toxic compound extrusion (MATE) transporter in *Nicotiana tabacum*. Proceedings of the National Academy of Sciences, USA 106, 2447–2452. 10.1073/pnas.0812512106 PMC2650162 19168636

[CIT0028] Moussaieff A Rogachev I Brodsky L Malitsky S Toal TW Belcher H Yativ M Brady SM Benfey PN Aharoni A 2013 High-resolution metabolic mapping of cell types in plant roots. Proceedings of the National Academy of Sciences, USA 110, E1232–E1241. 10.1073/pnas.1302019110 PMC3612672 23476065

[CIT0029] Mustroph A Zanetti ME Jang CJH Holtan HE Repetti PP Galbraith DW Girke T Bailey-Serres J 2009 Profiling translatomes of discrete cell populations resolves altered cellular priorities during hypoxia in Arabidopsis. Proceedings of the National Academy of Sciences, USA 106, 18843–18848. 10.1073/pnas.0906131106 PMC2764735 19843695

[CIT0030] Naur P Petersen BL Mikkelsen MD Bak S Rasmussen H Olsen CE Halkier BA 2003 CYP83A1 and CYP83B1, two nonredundant cytochrome P450 enzymes metabolizing oximes in the biosynthesis of glucosinolates in Arabidopsis. Plant Physiology 133, 63–72. 12970475 10.1104/pp.102.019240 PMC196579

[CIT0031] Ngala BM Haydock PPJ Woods S Back MA 2015 Biofumigation with *Brassica juncea*, *Raphanus sativus* and *Eruca sativa* for the management of field populations of the potato cyst nematode *Globodera pallida*. Pest Management Science 71, 759–769. 24965697 10.1002/ps.3849

[CIT0032] Nour-Eldin HH Andersen TG Burow M Madsen SR Jorgensen ME Olsen CE Dreyer I Hedrich R Geiger D Halkier BA 2012 NRT/PTR transporters are essential for translocation of glucosinolate defence compounds to seeds. Nature 488, 531–534. 22864417 10.1038/nature11285

[CIT0033] Nour-Eldin HH Hansen BG Norholm MHH Jensen JK Halkier BA 2006 Advancing uracil-excision based cloning towards an ideal technique for cloning PCR fragments. Nucleic Acids Research 34, e122. 17000637 10.1093/nar/gkl635 PMC1635280

[CIT0034] Sah H 1999 Stabilization of proteins against methylene chloride/water interface-induced denaturation and aggregation. Journal of Controlled Release 58, 143–151. 10053187 10.1016/s0168-3659(98)00148-5

[CIT0035] Sasse J Simon S Gubeli C Liu GW Cheng X Friml J Bouwmeester H Martinoia E Borghi L 2015 Asymmetric localizations of the ABC transporter PaPDR1 trace paths of directional strigolactone transport. Current Biology 25, 647–655. 25683808 10.1016/j.cub.2015.01.015

[CIT0036] Schreiner M Krumbein A Knorr D Smetanska I 2011 Enhanced glucosinolates in root exudates of *Brassica rapa* ssp *rapa* mediated by salicylic acid and methyl jasmonate. Journal of Agricultural and Food Chemistry 59, 1400–1405. 21265537 10.1021/jf103585s

[CIT0037] Strehmel N Böttcher C Schmidt S Scheel D 2014 Profiling of secondary metabolites in root exudates of *Arabidopsis thaliana*. Phytochemistry 108, 35–46. 25457500 10.1016/j.phytochem.2014.10.003

[CIT0038] Strehmel N Monchgesang S Herklotz S Kruger S Ziegler J Scheel D 2016 *Piriformospora indica* stimulates root metabolism of *Arabidopsis thaliana*. International Journal of Molecular Sciences 17, 1091. 10.3390/ijms17071091 PMC4964467 27399695

[CIT0039] Sugiyama A Shitan N Yazaki K 2007 Involvement of a soybean ATP-binding cassettetype transporter in the secretion of genistein, a signal flavonoid in legume–*Rhizobium* symbiosis. Plant Physiology 144, 2000–2008. 17556512 10.1104/pp.107.096727 PMC1949875

[CIT0040] Tsednee M Yang SC Lee DC Yeh KC 2014 Root-secreted nicotianamine from *Arabidopsis halleri* facilitates zinc hypertolerance by regulating zinc bioavailability. Plant Physiology 166, 839–U553. 25118254 10.1104/pp.114.241224 PMC4213112

[CIT0041] Weston LA Ryan PR Watt M 2012 Mechanisms for cellular transport and release of allelochemicals from plant roots into the rhizosphere. Journal of Experimental Botany 63, 3445–3454. 22378954 10.1093/jxb/ers054

[CIT0042] Witzel K Hanschen FS Klopsch R Ruppel S Schreiner M Grosch R 2015 *Verticillium longisporum* infection induces organ-specific glucosinolate degradation in *Arabidopsis thaliana*. Frontiers in Plant Sciencedoi: 10.3389/fpls.2015.00508. 10.3389/fpls.2015.00508 PMC4498036 26217360

[CIT0043] Zhuang XL Gao J Ma AZ Fu SL Zhuang GQ 2013 Bioactive molecules in soil ecosystems: masters of the underground. International Journal of Molecular Sciences 14, 8841–8868. 23615474 10.3390/ijms14058841 PMC3676760

[CIT0044] Ziegler J Facchini PJ 2008 Alkaloid biosynthesis: metabolism and trafficking. Annual Review of Plant Biology 59, 735–769. 10.1146/annurev.arplant.59.032607.092730 18251710

[CIT0045] Ziegler J Schmidt S Chutia R Muller J Bottcher C Strehmel N Scheel D Abel S 2016 Non-targeted profiling of semi-polar metabolites in Arabidopsis root exudates uncovers a role for coumarin secretion and lignification during the local response to phosphate limitation. Journal of Experimental Botany 67, 1421–1432. 26685189 10.1093/jxb/erv539 PMC4762384

