# Supplementary material for: Rhizosecretion of stele-synthesized glucosinolates and their catabolites requires GTR-mediated import in Arabidopsis
Source: J Exp Bot. 2016 Oct 4;68(12):3205–14. doi: 10.1093/jxb/erw355 (PMC5853541; doi:10.1093/jxb/erw355)
Supplement: supplementary_figures_S1_S3_tables_S1_S2 [file erw355_suppl_supplementary_figures_s1_s3_tables_s1_s2.pdf]

Rhizosecretion of stele-synthesized glucosinolates and their catabolites requires GTR-mediated import in *Arabidopsis*

Deyang Xu[1], Franziska S. Hanschen[2], Katja Witzel[3], Sebastian J. Nintemann[1], Hussam Hassan Nour-Eldin[1], Monika Schreiner[2] and Barbara Ann Halkier[1]*

Fig. S1. Sampling and profiling of GLS and their catabolites in *Arabidopsis* root exudates. (A) Single plant grown in sand filled pots for four weeks. (B) Morphology of 6-week-old sand grown Col-0 plants. Sand was carefully removed from roots and the plant ready for sampling root exudates. (C) Sampling root exudates. The plants were transferred to tubes filled with distilled water. (D) The recovery rate of external GLS standard 2-propenyl GLS and its corresponding catabolite allyl isothiocyanate (AITC) from *Arabidopsis* Col-0 root exudation.

Fig. S2. GLS concentration of rosettes and roots of 6-week-old, sand-grown *Arabidopsis* wildtype (Col-0) and *gtr1 gtr2*. GLS are grouped into total, indole, short-chained (SC) aliphatic (C3-C5), and long-chained (LC) aliphatic (C6-C8) GLS. For individual GLS data see Table S1. Error bars are SE (n = 10). **indicates statistically significant differences of *gtr1 gtr2* dKO plants compared to equivalent Col-0 Plants (two-tailed Students t-test, $P < 0.05$).

Fig. S3. Cell-type-specific expression of *MAM3*, *FMOGS-OX1*, *TGG4* and *TGG5*. Cell-type-specific expression for these genes was derived from microarray studies of RNA bound to ribosomes which were immuno-precipitated by use of epitope-tagged ribosomal protein from seedlings (Mustroph *et al.*, 2009)(http://efp.ucr.edu/).

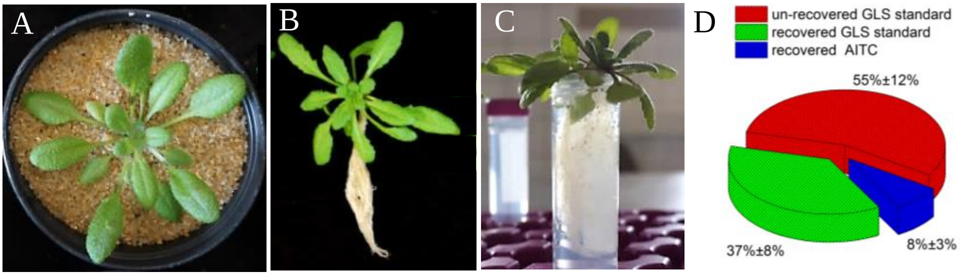

Fig. S1. Sampling and profiling of GLS and their catabolites in *Arabidopsis* root exudates. (A) Single plant grown in sand filled pots for four weeks. (B) Morphology of 6-week-old sand grown Col-0 plants. Sand was carefully removed from roots and the plant ready for sampling root exudates. (C) Sampling root exudates. The plants were transferred to tubes filled with distilled water. (D) The recovery rate of external GLS standard 2-propenyl GLS and its corresponding degradation product allyl isothiocyanate (AITC) from *Arabidopsis* Col-0 root exudation.

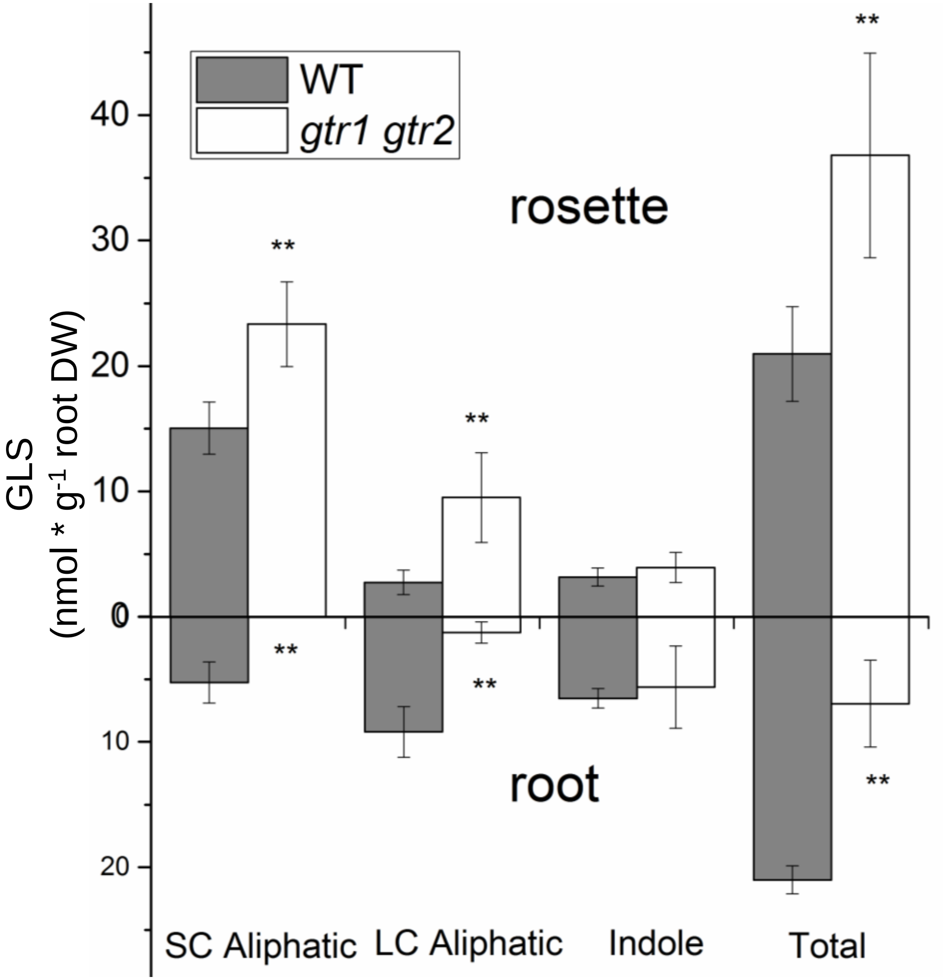

Fig. S2. GLS concentration of rosettes and roots of 6-week-old sand-grown *Arabidopsis* wildtype (Col-0) and *gtr1gtr2*. GLS are grouped into total, indole, short-chained (SC) aliphatic (C3-C5), and long-chained (LC) aliphatic (C6-C8) GLS. For individual GLS data see Table S1. Error bars are SE (n = 10). **indicates statistically significant differences of *gtr1gtr2* dKO plants compared to equivalent Col-0 Plants (two-tailed Students t-test, $P < 0.05$).

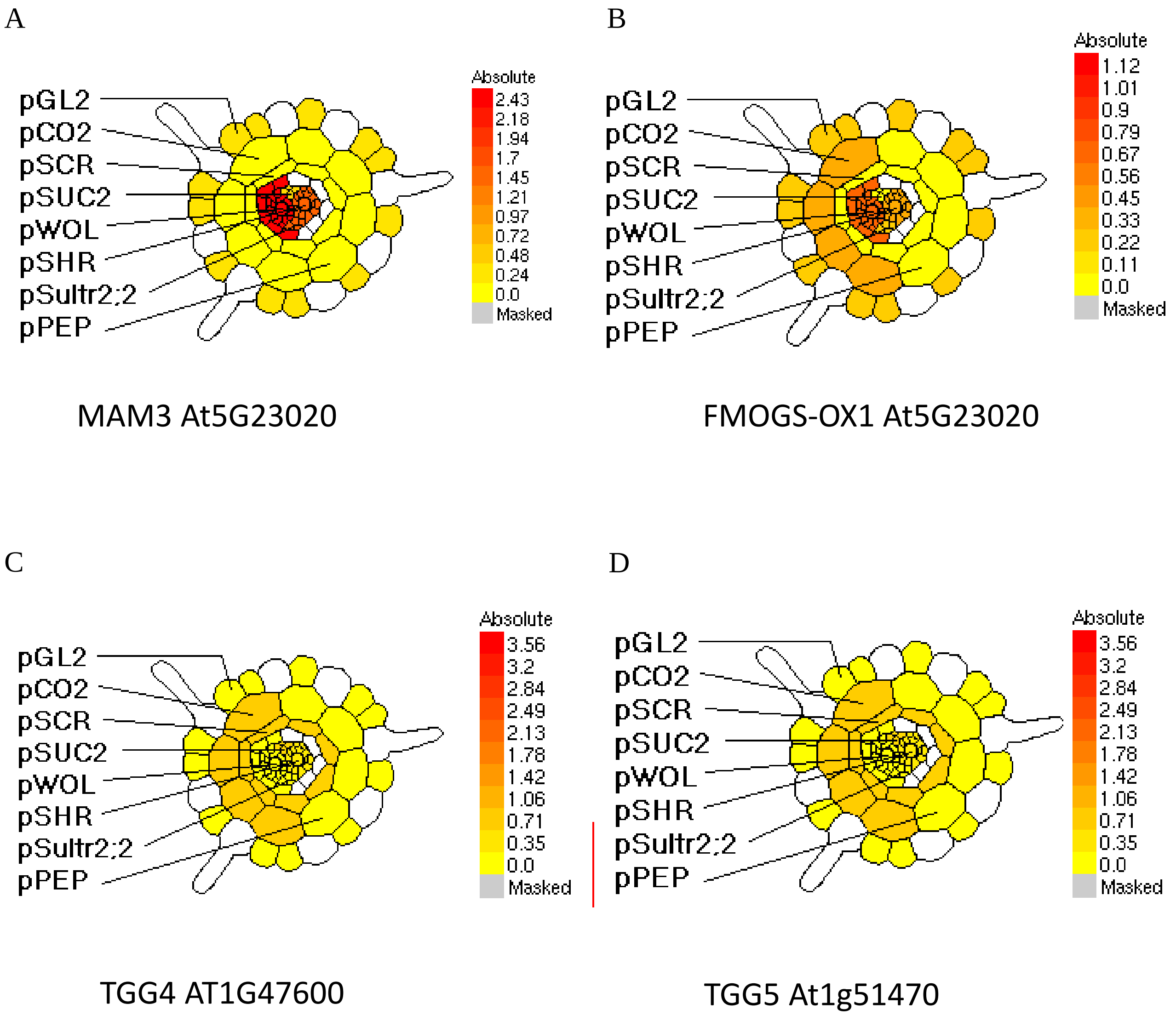

Fig. S3. Cell-type-specific expression of MAM3, FMOGS-OX1, TGG4 and TGG5. Cell-type-specific expression for these genes was derived from microarray studies of RNA bound to ribosomes which were immuno-precipitated by use of epitope-tagged ribosomal protein from seedlings (Mustroph et al., 2009)(http://efp.ucr.edu/).

Table S1 GLS present in the leaf and root of *Arabidopsis thaliana* Col-0 and the *gtr1 gtr2* mutant. Quantities shown in µmol g^{-1} dry weight, derived from the mean of three biological experiments and three batches of plants (each n=20 plants). Errors denote standard deviation. 4MTB: 4-(methylthio)butyl, 7MTH: 7-(methylthio)heptyl, 8MTO: 8-(methylthio)octyl, 3MSP: 3-(methylsulfinyl)propyl, 4MSB: 4-(methylsulfinyl)butyl, 7MSH: 7-(methylsulfinyl)heptyl, 8MSO: 8-(methylsulfinyl)octyl, I3M: 3-indolylmethyl, 4OHI3M: 4-hydroxy-3-indolylmethyl, 1MOI3M: 1-methoxy-3-indolylmethyl, 4MOI3M: 4-methoxy-3-indolylmethyl GLS. n.d. not detected. Significant differences between Col-0 and *gtr1 gtr2* mutant in the respective tissue comparison are indicated by asterisks (*: $P < 0.05$).

	Leaf		Root	
	Col-0	GTR1/2	Col-0	GRT1/2
Methylthioalkyl GLS				
4MTB	1.37±0.30	1.72±0.68	0.87±0.32	n.d.
7MTH	0.05±0.10	0.35±0.19	0.74±0.37	0.03±0.05*
8MTO	0.22±0.08	0.97±0.43*	4.35±1.95	0.62±0.47*
Methylsulfinylalkyl GLS				
3MSP	1.63±0.19	2.60±0.32*	0.58±0.15	0.01±0.02*
4MSB	12.05±1.59	19.02±2.37*	4.24±1.01	n.d.
7MSH	0.24±0.21	0.70±0.71	0.42±0.36	0.10±0.14
8MSO	2.24±0.58	7.50±2.25*	3.92±1.09	0.76±0.09*
Indole GLS				
I3M	2.17±0.34	3.12±0.80	1.53±0.19	0.84±0.10*
4OHI3M	0.00±0.01	n.d.	0.41±0.03	0.10±0.05*
1MOI3M	0.26±0.20	0.34±0.19	4.79±1.68	4.93±2.62
4MOI3M	0.74±0.18	0.47±0.21	1.02±0.48	0.75±0.38

Table S2 Glucosinolate catabolites present in the leaf and root of *Arabidopsis thaliana* Col-0 and the *gtr1 gtr2* mutant. Quantities shown in µmol g^{-1} fresh weight, derived from the mean of three biological experiments and three batches of plants (each n=20). Errors denote standard deviation. 4MTB-CN: 5-(methylthio)penylnitrile, 4MTB-ITC: 4-(methylthio)butyl ITC, 5MTP-ITC: 5-(methylthio)pentyl ITC, 6MTH-ITC: 6-(methylthio)hexyl ITC, 7MTH-CN: 8-(methylthio)octylnitrile, 7MTH-ITC: 7-(methylthio)heptyl ITC, 8MTO-CN: 9-(methylthio)nonylnitrile, 8MTO-ITC: 8-(methylthio)octyl ITC, 3MSP-ITC: 3-(methylsulfinyl)propyl ITC, 4MSB-CN: 5-(methylsulfinyl)pentylnitrile, 4MSB-ITC: 4-(methylsulfinyl)butyl ITC, 8MSO-ITC: 8-(methylsulfinyl)octyl ITC, IAN: indole-3-acetonitrile; 1-Methoxy-IAN; 1-methoxyindole-3-acetonitrile; 4-Methoxy-IAN: 4-methoxyindole-3-acetonitrile. n.d. not detected. Significant differences between Col-0 and *gtr1 gtr2* mutant in the respective tissue comparison are indicated by asterisks (*: $p<0.05$).

	Leaf		Root	
	Col-0	GTR1/2	Col-0	GTR1/2
Methylthioalkyl catabolites				
4MTB-CN	n.d.	n.d.	0.01±0.01	n.d.
4MTB-ITC	0.08±0.06	0.10±0.04	0.03±0.01	n.d.
5MTP-ITC	0.01±0.01	0.01±0.01	0.00±0.01	n.d.
6MTH-ITC	n.d.	n.d.	0.01±0.00	n.d.
7MTH-CN	n.d.	n.d.	0.01±0.00	n.d.
7MTH-ITC	0.01±0.01	0.04±0.02	0.06±0.02	0.01±0.01*
8MTO-CN	n.d.	n.d.	0.11±0.03	0.02±0.01*
8MTO-ITC	0.03±0.03	0.15±0.06*	0.54±0.20	0.06±0.03*
Methylsulfinylalkyl catabolites				
3MSP-ITC	0.10±0.09	0.17±0.13	0.01±0.01	n.d.
4MSB-CN	0.02±0.01	0.05±0.02	0.02±0.02	n.d.
4MSB-ITC	1.00±0.85	1.56±0.79	0.13±0.15	n.d.
8MSO-ITC	0.02±0.03	0.05±0.05	n.d.	n.d.
Indole catabolites				
IAN	n.d.	0.00±0.01	0.08±0.06	0.06±0.03
1-Methoxy-IAN	n.d.	0.32±0.55	0.04±0.04	0.01±0.01
4-Methoxy-IAN	n.d.	n.d.	0.01±0.00	0.01±0.00
